# N-Acetylaspartate Reduction in the Medial Prefrontal Cortex Following 8 weeks of Risperidone Treatment in First-Episode Drug-Naïve Schizophrenia Patients

**DOI:** 10.1038/srep09109

**Published:** 2015-03-16

**Authors:** Xiaofen Zong, Maolin Hu, Zongchang Li, Hongbao Cao, Ying He, Yanhui Liao, Jun Zhou, Deen Sang, Hongzeng Zhao, Jinsong Tang, Luxian Lv, Xiaogang Chen

**Affiliations:** 1 Institute of Mental Health, the Second Xiangya Hospital of Central South University, Changsha, Hunan, China; 2 Unit on Statistical Genomics, National Institute of Mental Health, NIH, Bethesda, USA; 3 Department of Radiology, The Second Affiliated Hospital of Xinxiang Medical University, Xinxiang, Henan, China; 4 Henan Key Lab of Biological Psychiatry, Xinxiang Medical University, Xinxiang, Henan, PR China; Department of Psychiatry, The Second Affiliated Hospital of Xinxiang Medical University, Xinxiang, Henan, China; 5 Key Laboratory of Psychiatry and Mental Health of Hunan Province, Central South University, Changsha, Hunan, China; 6 National Technology of Institute of Psychiatry, Central South University, Changsha, Hunan, China

## Abstract

It is unclear whether N-acetylaspartate (NAA) depletions documented in schizophrenia patients might be due to the disease progression or medications. Here we investigated longitudinal NAA changes in drug-naïve first-episode patients (FEP) who are relatively free from chronicity. Forty-two drug-naïve FEP and 38 controls were enrolled in this study to explore the effect of 8-week risperidone monotherapy on NAA. All spectra were obtained from the medial prefrontal cortex (MPFC) on a 3.0 T MRI and analyzed with LCModel. At baseline, patients presented no significant differences in NAA (P = 0.084) or NAA/Cr + Pcr (P = 0.500) compared to controls; NAA levels were negatively correlated with PANSS total scores (P = 0.001) and WCST-PE (P = 0.041). After treatment, patients demonstrated significant reductions of NAA (P < 0.001) and NAA/Cr + Pcr (P < 0.001), and significant improvement in PANSS-P (P < 0.001) and PANSS-G (P < 0.001) symptoms. We detected no significant correlations between NAA alterations and PANSS-P (P = 0.679) or PANSS-G (P = 0.668) symptom changes; nor did NAA/Cr + Pcr changes with alterations in PANSS-P (P = 0.677) and PANSS-G (P = 0.616). This is the first evidence that short-term risperidone treatment induces an acute reduction of MPFC NAA during the early phase of schizophrenia, which may be a previously unavailable biomarker to indicate risperidone with a similar pharmacological mechanism, although the functional significance is still unclear.

Schizophrenia (SZ) is a complicated mental disorder and characterized by a cluster of symptoms, such as positive and negative symptoms, cognitive deficits and so on. Recently, abnormalities of brain metabolites, such as bioenergetic molecules or neurotransmitters, have been an alternative perspective to explore the pathophysiology of SZ. The amino acid of N-acetylaspartate (NAA) has been widely investigated in many clinical studies^1^,^2^,^3^, as two primary factors bring it to the attention of the neuroscience. First is its exclusive localization in neurons (not in glial cells), which makes it one of the reliable markers of neuronal number or function^4^. The second is its prominent proton signal in proton magnetic resonance spectroscopy (^1^H-MRS), which makes it one of the most accurate and reliable markers for ^1^H-MRS researches^5^.

Cross-sectional ^1^H-MRS studies have shown consistent findings that chronic SZ patients have significant NAA depletion in the prefrontal cortex (PFC)^6^,^7^, while several other studies didnot observe NAA anomalies in the PFC in the early phase of SZ^8^,^9^,^10^. In one recent study, Natsubori et al^11^ investigated the subjects at three different stages of illness including chronic patients, FEP and ultrahigh risk populations, and found that significant reduction of NAA in the medial PFC (MPFC) exists specifically in chronic subjects but not in FEP or ultrahigh risk ones. Consistently, Ohrmann et al^12^ suggested that chronic patients but not FEP have NAA declines in the PFC. These may reflect the progressive alterations of neurometabolites in SZ^13^. However, besides the disease itself, many other factors including treatment effects may also affect the NAA deficits observed in chronic SZ through cross-sectional studies.

In the pharmacological properties of neuroleptics, D2 receptor antagonism plays a key role in alleviating patients' positive symptoms, although, the relative success is limited by the lack of efficacy for negative symptoms and cognitive impairments^14^. The severity of negative symptoms and cognitive impairments of SZ patients is related to the reduction of D1 receptors in the PFC^15^. Unfortunately, blockade of D2 receptor^16^ or antipsychotics exposure^17^ can induce down-regulation of D1 receptors in the PFC. Additionally, it has been speculated that the mitochondria effects of antipsychotics reported broadly^18^,^19^,^20^ are possibly regulated by downstream effects following blockade of relative dopamine receptors in the cortex^21^. Moreover, D1 receptor agonists can partially prevent neuroleptic-induced deleterious influence on mitochondrial function^22^. Importantly, NAA is exclusively synthesized in neuronal mitochondria and mitochondrial impairments can rapidly affect NAA production^23^.

Longitudinal designs can partly overcome the unresolved problem in cross-sectional studies and relatively distinguish between the progressive pathogenesis and the treatment effects. However, recently, longitudinal ^1^H-MRS studies investigating the influence of neuroleptics on central NAA have yielded inconsistent findings^8^,^24^,^25^. Most studies have used chronically treated patients^1^,^25^,^26^ or minimally treated patients^8^,^24^, and the few studies of drug-naïve patients^2^ are limited by medication with heterogeneous drugs. Therefore, the confounders of medications and chronicity can not be completely excluded and the effect of antipsychotics on NAA still has to be explored. First-episode and drug-naïve SZ patients are in the very early phase of illness when relatively ‘free' from prior treatment and chronicity. To date, there is still lack of longitudinal studies with large samples of drug-naïve first-episode patients (FEP) to investigate NAA alterations after short-term monotherapy.

Therefore, we performed this longitudinal study of ^1^H-MRS in a relatively large cohort of drug-naïve FEP to explore the NAA changes after 8-week treatment withrisperidone monotherapy. We selected MPFC as the voxel of interest. We test the hypothesis that first-episode SZ patients would have alterations in NAA levels after treatment. We also conducted an exploratory research to analyse whether NAA changes would be relevant to alterations in clinical symptoms and cognitive function.

## Results

### Demographic and clinical characteristics between cases and controls at Baseline

Forty-two patients met the criteria of drug-naïve first-episode SZ (duration of illness = 8.38 ± 2.61 months). As shown in table 1, patient and control groups were well matched in age, gender, education, handedness, and alcohol and tobaco usage (Ps > 0.05). However, significant lower performance in WCST categories (t_78_ = 2.718, P = 0.008) and WCST persererative errors (WCST-PE) (t_78_ = 9.041, P < 0.001) were observed in case group.

### Longitudinal comparisons of clinical symptoms and cognitive function between baseline and week 8

As shown in table 1, we observed significant decreases in PANSS total scores (PANSS-T) (t_41_ = 13.19, P < 0.001), PANSS positive symptom scores (PANSS-P) (t_41_ = 14.58, P < 0.001) and PANSS general psychopathological symptom scores (PANSS-G) (t_41_ = 13.37, P < 0.001), while no significant changes in PANSS-N (t_41_ = 1.42, P = 0.163) after treatment. We detected no significant changes in WCST categories (t_41_ = 1.46, P = 0.151) and WCST-PE (t_41_ = 1.72, P = 0.093) after treatment. Therefore changes of PANSS-N and WCST performance were not further calculated with the Pearson correlation analysis.

### Quality of ^1^H-MRS spectra

The mean ± SD measures of FWHM from LCModel were 0.065 ± 0.017 ppm for patients at baseline, 0.060 ± 0.018 ppm for patients at 8-week follow-up, and 0.067 ± 0.018 ppm for controls. The mean ± SD measures of S/N from LCModel were 21.36 ± 2.35 for patients at baseline, 20.03 ± 2.36 for patients at 8-week follow-up, and 20.58 ± 3.23 for controls. There were no significant differences in both of the two measures in the three groups: F_2_ = 1.492, P = 0.229 for FWHM; and F_2_ = 2.510, P = 0.086 for S/N. The range of Cramer–Rao minimum variance of NAA was from 1% to 10% in the three groups.

### Comparison of baseline NAA and NAA/Cr + Pcr between cases and controls and longitudinal changes following treatment

**Figure 1:**
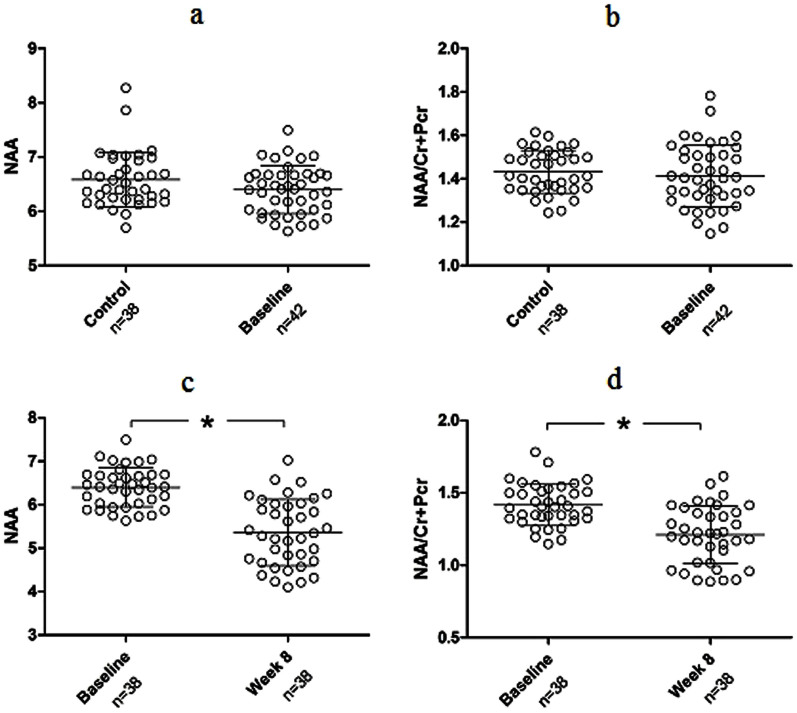
Medial prefrontal cortex NAA levels and NAA/Cr + Pcr ratio of each subject in schizophrenia patients and controls at baseline and after 8-week treatment. (a) At baseline patients had no significant difference in NAA levels compared to controls (t_78_ = 1.75, P = 0.084, independent-samples t tests). (b) At baseline patients had no significant difference in NAA/Cr + Pcr compared to controls (t_78_ = 0.68, P = 0.500 independent-samples t tests). (c) Patients had significant reductions in NAA levels following 8-week treatment (t_37_ = 6.98, P < 0.001, paired-samples t tests). (d) Patients had a significant reduction in NAA/Cr after 8-week treatment (t_37_ = 5.38, P < 0.001, paired-samples t tests). *statistical levels of significance.

At baseline, no significant differences were observed between patients and controls in terms of NAA levels (t_78_ = 1.751, P = 0.084, figure 1a), NAA/Cr + Pcr (t_78_ = 0.677, P = 0.500, figure 1b) and Cr + Pcr (t_78_ = 0.541, P = 0.590). After 8-week treatment, there were significant declines in NAA levels (t_37_ = 5.187, P < 0.001, figure 1c) and NAA/Cr + Pcr (t_37_ = 4.097, P < 0.001, figure 1d) in patients, but no significant alterations of Cr + Pcr levels (t_37_ = 1.210, P = 0.234). Moreover, responding patients showed no differences in baseline NAA, NAA/Cr + Pcr or Cr + Pcr (Ps > 0.05) relative to non-responding patients (details shown in Table S1). After treatment, the changes of NAA or NAA/Cr + Pcr demonstrated no significant differences between responding and non-responding patients (Ps > 0.05) (Table S2).

### Correlations of Baseline NAA or NAA/Cr + Pcr with Clinical and Demographic Characteristics in Patients

**Figure 2:**
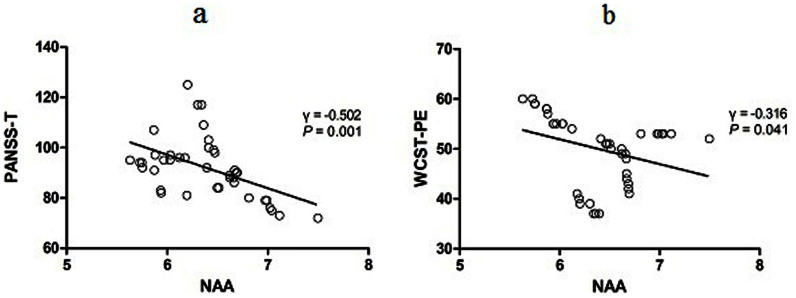
Correlations of NAA levels with clinical symptoms or cognitive function in patients at baseline. (a) NAA levels were negatively related to PANSS total scores in 42 patients at baseline (γ = −0.502, P = 0.001). (b) NAA levels were negatively correlated with Wisconsin Card Sorting Test– perseveration errors in 42 patients at baseline (γ = −0.316, P = 0.041). Abbreviations: PANSS–T, PANSS total scores; WCST–PE, Wisconsin Card Sorting Test–perseveration errors.

At baseline, we noted significantly negative correlations of NAA levels with PANSS-total scores (γ = −0.502, P = 0.001, Figure 2a) and WCST-PE (γ = −0.316, P = 0.041, Figure 2b). There were no significant correlations between NAA levels and PANSS-P, PANSS-G or PANSS-N (Ps > 0.05). NAA/Cr + Pcr was not related to PANSS-T, PANSS-P, PANSS-G, PANSS-N or WCST-PE (Ps > 0.05). Moreover, NAA levels or NAA/Cr + Pcr didnot show significant correlatioins with age, education and illness duration prior to treatment (Ps > 0.05). Statistical analysis also indicated that NAA levels or NAA/Cr + Pcr were not influenced by gender (t_40_ = −0.156, P = 0.876; t_40_ = 0.895, P = 0.376, respectively), tobaco use (t_40_ = −0.092, P = 0.927; t_40_ = −1.132, P = 0.264, respectively), alcohol use (t_40_ = −0.413, P = 0.681; t_40_ = −0.646, P = 0.522, respectively), and family history (t_40_ = 1.746, P = 0.088; t_40_ = 1.195, P = 0.239, respectively). We showed the distribution of the NAA levels cross education and age in both patients and controls in Figure S1.

### Correlations of NAA alterations with symptom changes after treatment

After 8-week treatment, we observed no significant correlations between changes in NAA levels and alterations in PANSS-T (PANSS reductive ratio) (γ = −0.289, P = 0.088), PANSS-P (γ = −0.238, P = 0.162) and PANSS-G (γ = −0.278, P = 0.101). Nor did changes in NAA/Cr + Pcr with alterations in PANSS-T (γ = −0.255, P = 0.134), PANSS-P (γ = −0.145, P = 0.400) and PANSS-G (γ = −0.291, P = 0.094). In addition, acute alterations of NAA levels and NAA/Cr + Pcr following drug treatment were not influenced by age (γ = 0.145, P = 0.384; γ = −0.230, P = 0.165, respectively), education (γ = −0.072, P = 0.669; γ = 0.045, P = 0.788, respectively), illness duration prior to treatment (γ = 0.179, P = 0.283; γ = 0.093, P = 0.580, respectively), gender (t_36_ = −0.332, P = 0.742; t_36_ = −0.549, P = 0.586, respectively), tobaco use (t_36_ = 0.453, P = 0.662; t_36_ = 1.137, P = 0.263, respectively) and alcohol use (t_36_ = 1.692, P = 0.099; t_36_ = 1.313, P = 0.198, respectively).

## Discussion

The key finding of our present study was that there was an acute decline of MPFC NAA and NAA/Cr + Pcr after 8-week risperidone exposure in drug-naïve FEP. To our knowledge, this study provides the first evidence that short-term risperidone treatment induces reductions of NAA in the MPFC in the early phase of SZ. Importantly, we found no significant correlations of the baseline NAA and NAA/Cr + Pcr with the illness duration, and changes of NAA levels and the NAA/Cr following treatment were not affected by the illness duration. These results indicated that the acute NAA decline was more likely to be the result of medications rather than the disease progression. Moreover, patients have no significant differences in the baseline NAA and NAA/Cr + Pcr compared to controls, and they have experienced psychotic symptoms for 8.38 ± 2.61 months. Therefore, the treatment effect is more reasonable to explain for the acute NAA depletion.

Recently, Lieberman et al^13^ completed the first meta-analysis of ^1^H-MRS findings, comparing different brain regions in individuals from prodrome to chronic SZ (97 studies: 2477 chronic patients, 923 FEP, 392 individuals at high risk of SZ, and 3103 controls). Concurring with our present results, for FEP in the frontal lobe, illness duration presents no significant influence on NAA; while significant effects of antipsychotic medications on NAA were found (lower NAA levels with longer duration of medications). Their findings supported the hypothesis that the antipsychotic treatment rather than the illness progression is associated with the decreased frontal NAA in FEP.

Combining with the mounting available evidence^23^,^27^,^28^, the acute NAA decline after medications in this study may be a marker of reversible neuronal mitochondrial dysfunction, rather than permanent apoptosis. The NAA depletion in our study would be better understood in the context of pharmacologic properties of risperidone. Although risperidone has low affinity for D1 receptors^29^, exposure to risperidone can induce approximately two-thirds reduction of D1 receptors expression in primates PFC^17^. Furthermore, it has reported that D1 rather than D2 receptor agonists, can partially prevent neuroleptic-induced deleterious influence on the function of neuronal mitochondria^22^. Thus, it is possible that the adverse effect of risperidone on NAA depletions in the MPFC presented in this study may caused by, at least partly, a drug-related D1 receptors down-regulation in the MPFC. Additionally, unlike haloperidol, risperidone firstly inhibites neuronal dyfunction in the frontal cortex, but not in the extrapyramidal regions^21^, which consists with our present findings that the MPFC NAA decline occured in the absence of extrapyramidal adverse effects.

In this study, patients were significantly benefited from medications in terms of improved positive symptoms. This may be due to the fact that D2 receptor antagonism of risperidone plays a key role in alleviating patients' positive symptoms. However, their negative symptoms and poor performance of WCST were not improved. Positron emission tomography (PET) study demonstrated that reductions of PFC D1 receptor in drug-free or drug-naïve SZ patients are significantly related to their negative symptoms and poor performance of WCST^15^. In this case, risperidone-elicited down-regulation of D1 receptor might further exacerbate patients' baseline negative symptoms and poor performance of WCST, which appears to contradict our findings of no changes in negative symptoms and WCST performance. However, we still can't fully rule out the possibility that patients' negative symptoms and impaired cognitive function would be further aggravated if medications had been prolonged and that the aggravation might be related to decreased NAA. This possibility is supported by the important findings in the meta-analysis of Lieberman et al^13^ that lower NAA is related to higher negative symptom scores in chronically treated patients. Additionally, since the risperidone effect on D1 receptor down-expression may be not related to alterations of positive symptoms, in this study the NAA changes were not related to the alterations of positive symptoms. Therefore, NAA depletions may be secondary to treatment-related down-regulation of D1 receptor in the MPFC.

Our findings of early NAA defecits after risperidone medications stand in contrast to the available clinical ^1^H-MRS researches that have reported a modest increase^1^,^2^,^26^,^30^,^31^ or no changes^1^,^24^ in NAA levels or NAA/Cr after treatment with antipsychotics^32^, including risperidone. The discrepancy may be due to several factors. First, sample variations, such as sample sizes, ethnicity, symptoms severity, length of untreated psychotic symptoms and so on, may contribute to inconsistent findings. Second, according to the speculation that the risperidone-related down-regulation of D1 receptors in the MPFC may have detrimental effects on neuronal NAA, different antipsychotics may bring discrepant effects on metabolite levels in the same brain area due to different pharmacological properties. Therefore it seems appropriate for future studies to investigate the effect of each drug separately in large samples of drug-naïve patients. Third, and perhaps most important, different lengths of follow-up may lead to an artificial discrepancy and heterogeneity. If NAA levels are influenced independently by antipsychotics but not the disease progression (although to date it is difficult to absolutely exclude the effects of illness itself), we speculate that there appears to be a nonlinear correlation between the antipsychotic exposure and NAA levels. If drug-induced NAA alterations occur very early (eg. 8–12 weeks), shorter or longer follow-ups in subjects may obscure the antipsychotic effects on NAA levels, namely, patients followed longer may have less or no NAA reductions because the NAA decline may occur earlier.

Another finding in this study is that the baseline NAA level was negatively correlated with PANSS-T and WCST-PE, namely, higher NAA levels may be beneficial for patients to experience less serious symptoms and cognitive impairment, which consists with broad studies^12^,^25^,^31^,^32^,^33^. However, in this case, decreased NAA levels after treatment would have been related to exacerbated clinical presentations, but in this study patients' PANSS-T symptoms improved and their poor performance of WCST remained stable. Furthermore, the alterations of NAA or NAA/Cr + Pcr after treatment showed no significant differences between responding and non-responding patients. On integrating our present results with that of Szulc et al^25^, Ertugrul et al^31^, and Bustillo et al^8^, the evidence suggests that the marked reduction of the MPFC NAA following the risperidone treatment may be a previously unavailable biomarker to indicate the antipsychotic drugs with a similar pharmacological mechanism, although the functional significance is still unclear.

Additionally, patients had no significant differences in baseline NAA and NAA/Cr + Pcr in the MPFC compared to controls. Cross-sectional ^1^H-MRS studies have broadly found NAA reductions in the thalamus, hippocampus and dorsolateral PFC primarily in chronic patients, but also in FEP, however, changes of MPFC NAA in SZ yet seem to be inconclusive^6^. Whether SZ patients exist abnormalities of MPFC NAA should be further explored.

Certain limitations of our present study need considerations. First, even though drug-naïve FEP are in the very early phase of illness when “free” from chronicity relatively, the influence of the potential progressive characteristic of SZ on NAA depletions can not be fully excluded. Second, due to the absence of data about the brain metabolite alterations after medication withdrawal or the relative parallel research design of neuroprotective therapies, we can't absolutely clarify whether the early acute NAA defecit is a marker of neuron apaptosis or dysfunction^6^. Third, although, to date, this is the largest longitudinal study in first-episode drug-naïve patients given risperidone monotherapy, the sample size perhaps still lacks power to completely eliminate the possibility that the NAA change is an acute phe-random and not systematically affect one group or the other.

In conclusion, our present findings demonstrated a possible evidence that risperidone monotherapy induces significant NAA depletions in the MPFC in the early phase of SZ, although a potential effect of the disease progression still cannot be excluded completely. Our findings may shed light on the nature of detrimental effect of risperidone on neuronal function during the early phase of SZ. We underscore the need for longer follow-up to examine whether negative symptoms and cognitive function of SZ patients would be further exacerbated and whether the exacerbation would be related to NAA depletions following prolonged medications. Additionally, our observations may indicate that MPFC NAA is a biomarker of baseline symptom severity and cognitive function.

## Methods

### Participants

Forty-two in-patients were recruited from August 2012 to December 2013 at the Second Affiliated Hospital, Xinxiang Medical University, Xinxiang, Henan, China. All patients were drug-naïve and were experiencing their first-episode psychosis when enrolled to this study. They were diagnosed by one of two trained psychiatrists according to the Structured Clinical Interview for DSM-IV-TR (Diagnostic and Statistical Manual of Mental Disorders, forth edition, American Psychiatric Association, 2000), patient version (SCID-I/P), and all patients met the diagnostic criteria for SZ. Thirty-five patients had been diagnosed as paranoid schizophrenia, while 7 patients undifferentiated schizophrenia. The patients had an onset age from 18 to 45 years, and had less than 1 year duration of disease. Thirty-eight gender-, age-, and education-matched healthy controls were recruited by advertisement. All controls were screened for lifetime absence of psychiatric diseases by using the SCID, Non-Patient Edition, and were confirmed to be without a family history of psychosis in their first-degree relatives. All participants were Chinese Han people and right-handed. Participants with a history of neurological illness, physical disorders or substance abuse were excluded from this study.

This study was approved by the Ethics Committee of the Second Xiangya Hospital of Central South University (No. S088, 2012), and the cooperated hospital, the Second Affiliated Hospital of Xinxiang Medical University. This study was carried out in accordance with the Declaration of Helsinki. Signed informed consent forms were obtained from all participants. All patients were aware that they were entitled to withdraw from this study at any time without jeopardizing their clinical treatment. Four patients did withdraw at the follow-up ^1^H-MRS scanning, but they have completed clinical assessment.

### Medication

All patients took risperidone monotherapy for 8 weeks. Concomitant treatment with mood stabilizers or antidepressants was not permitted. The dose titration was according to the clinical status of patients. Compliance with risperidone was monitored by clinical interviews once a week. No serious side effects were reported during the treatment.

### ^1^H-MRS scanning procedure

**Figure 3:**
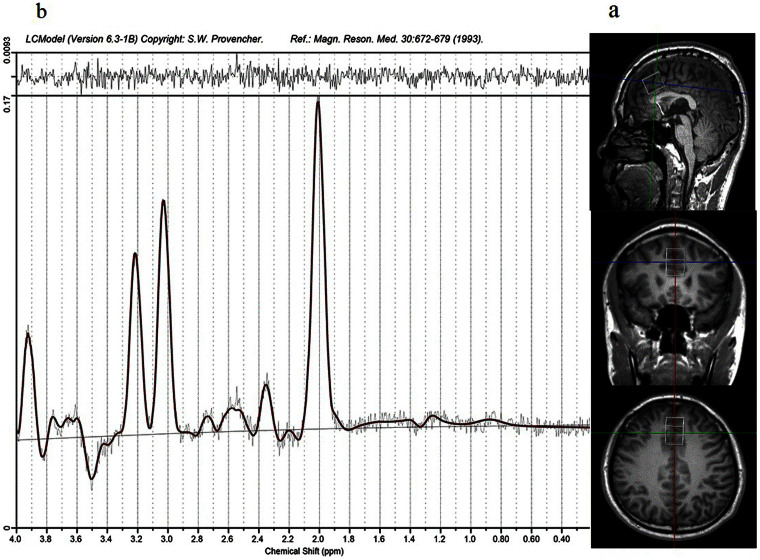
Spectroscopic voxel placement and proton magnetic resonance (^1^H-MRS) spectra. (a) Voxel placement in medial prefrontal cortex (MPFC). (b) Sample ^1^H-MRS spectra acquired from MPFC as fitted by LCModel (provencher, 1993).

All patients had ^1^H-MRS scans within 24 h after included in this study when they were free from drugs, and scans were scheduled again for patients after 8-week treatment, while controls only had baseline scans. All examinations were completed on a 3.0 T magnetic resonance imager (Siemens, Verio, Germany) using a 16-channel head coil at the Magnetic Imaging Centre of the Second Affiliated Hospital of Xinxiang Medical University. Participants were fitted with earplugs and foam pads to reduce scanner noise and limit head motion. T1-weighted anatomical images were obtained with 3-dimensional rapid acquisition with gradient echo for voxel tissue segmentation (TR 1900 ms; TE 2.52 ms; FOV 250 × 250 mm; flip angle 9°; slice thickness 1 mm; gap 0 mm; NEX 1; slices 176). ^1^H-MRS spectra were acquired using the standard PRESS sequence (svs_se, TR 8s, TE 80 ms, NEX 128). A 20 × 20 × 25 mm voxel of interesting (VOI) was prescribed to include mostly gray matter in the medial prefrontal cortex using coronal, sagittal and transverse images (Figure 3). The unsuppressed water spectra were also scanned in the same voxel. The priliminary quality detection of spectra was performed with the spectroscopic processing of Siemens Spectroscopy application (Syngo B17, Germany). Spectra with apparent baseline drift were excluded and scanning would be repeated again after patients' consent was obtained.

NAA was quantified using LCModel version 6.3-1B (LCMODEL Inc. CA) at the Second Affiliated Hospital, Shantou University Medical College, Shantou, Guangdong. The cerebrospinal fluid (CSF) content in the MPFC was served as the internal standard for calculation of absolute concentration of NAA. NAA scaled to creatine plus phosphocreatine (NAA/Cr + Pcr) was also selected as an important value reported by LCModel, as it is also a helpful measure of neuronal integrity^34^. To ensure data quality and avoid bias, any spectra that met one or more of these criteria was discarded: (1) the Cramer–Rao minimum variance > 15%; (2) NAA FWHM < 0.1; and (3) the signal-to-noise ratio (S/N) > 10. More details are available in the LCModel manual (http://s-provencher.com/pages/lcm-manual.shtml).

### Clinical assessments

Patients (both at baseline and week 8) and controls (only at baseline) completed computerized 128-card version of the Wisconsin Card Sorting Test (WCST), which can evaluate subjects' executive function of frontal cortex, including perseveration, flexibility and abstract problem solving strategies^35^. Both at baseline and week 8, symptoms severity of patients was measured by using the 30-item Positive and Negative Syndrome Scale (PANSS)^36^.

### Statistical analyses

Statistical analyses were completed using SPSS 13.0 (SPSS Inc., USA). Group differences of demographic data, metabolites levels and WCST scores were performed using independent-samples t tests or χ^2^ square test. Clinical symptom scores and WCST performance of patients between the two time points (baseline and week 8) were performed using paired-samples t tests. Correlations between alterations in brain metabolites and changes in clinical symptoms or WCST scores were further calculated with the partial correlation analysis (with baseline symptom scores or metabolite levels as unconcerned confounding factors) only if there was a significant variation between the two time points. The significance level was set with a threshold of P < 0.05.

## Supplementary Material

Supplementary Information Supplementary Information

## Figures and Tables

**Table 1:** Demographic and Clinical Characteristics of Controls and Schizophrenia Patients for Baseline and Follow-up

Data	Control (n = 38)	Patient (n = 42)		t/*χ*^2^	*P*
		Baseline	Week 8		
Age, mean ± SD,years	24.76 ± 4.56	24.86 ± 4.80*		0.09	0.929
Gender(male/female)	25/13	27/15*		0.02	0.888
Education, mean ± SD, years	11.05 ± 2.91	10.48 ± 2.84*		0.90	0.373
Handedness (right/left)	38/0	42/0*			-
Alcohol use(y/n)	9/29	6/36*		1.16	0.282
Tobacco use(y/n)	8/30	9/33*		0.002	0.967
Family history (y/n)	0/38	15/27*			
PANSS-T		91.90 ± 11.23	67.24 ± 10.10**	13.19	<0.001
PANSS-P		25.60 ± 3.75	15.83 ± 3.28**	14.58	<0.001
PANSS-N		18.17 ± 5.21	17.07 ± 4.86**	1.42	0.163
PANSS-G		48.14 ± 6.46	34.33 ± 4.71**	13.37	<0.001
WCST-categories	4.53 ± 1.06	3.95 ± 0.82*	3.64 ± 1.16	2.72	0.008
WCST-PE	32.00 ± 10.35	49.95 ± 6.88*	47.02 ± 9.87	9.04	<0.001

Abbreviations: PANSS, Positive and Negative Symptoms Scale; PANSS-T, PANSS total scores; PANSS-P, PANSS positive symptom scores; PANSS-N, PANSS negative symptom scores; PANSS-G, PANSS general psychopathological symptom scores; WCST, Wisconsin Card Sorting Test; WCST-PE, WCST perseveration errors; *vs. controls; **vs. baseline.
