# Supplementary material for: N-Acetylaspartate Reduction in the Medial Prefrontal Cortex Following 8 weeks of Risperidone Treatment in First-Episode Drug-Naïve Schizophrenia Patients
Source: Sci Rep. 2015 Mar 16;5:9109. doi: 10.1038/srep09109 (PMC4894446; doi:10.1038/srep09109)
Supplement: Supplementary Information [file srep09109-s1.docx]

N-Acetylaspartate Reduction in the Medial Prefrontal Cortex Following 8 weeks of Risperidone Treatment in First-Episode Drug-Naïve Schizophrenia Patients

Xiaofen Zong^1^, Maolin Hu^1^, Zongchang Li^1^, Hongbao Cao^2^, Ying He^1^, Yanhui Liao^1^, Jun Zhou^1^, Deen Sang^3^, Hongzeng Zhao^3^, Jinsong Tang^1*^, Luxian Lv^4*^, Xiaogang Chen^1,5,6*^

1 Institute of Mental Health, the Second Xiangya Hospital of Central South University, Changsha, Hunan, China.

2 Unit on Statistical Genomics, National Institute of Mental Health, NIH, Bethesda, USA.

3 Department of Radiology, The Second Affiliated Hospital of Xinxiang Medical University, Xinxiang, Henan, China.

4 Henan Key Lab of Biological Psychiatry, Xinxiang Medical University, Xinxiang, Henan, PR China; Department of Psychiatry, The Second Affiliated Hospital of Xinxiang Medical University, Xinxiang, Henan, China.

5 Key Laboratory of Psychiatry and Mental Health of Hunan Province, Central South University, Changsha, Hunan, China.

6 National Technology of Institute of Psychiatry, Central South University, Changsha, Hunan, China.

*Corresponding author:

Xiaogang Chen M.D. Ph.D., Fax: +86-731-8553-1571, Telephone:+86-731-8553-1571, Email: chenxghn@gmail.com

Correspondence may also be addressed to:

Jinsong Tang M.D. Ph.D., Fax: +86-731-8553-1571, Telephone: +86-731-8553-1571, E-mail: tangjinsonghn@gmail.com

Luxian Lv M.D. Ph.D., Fax:+86-373-337-3906, Telephone: +86-373-337-3906, Email: lvluxian@126.com

**
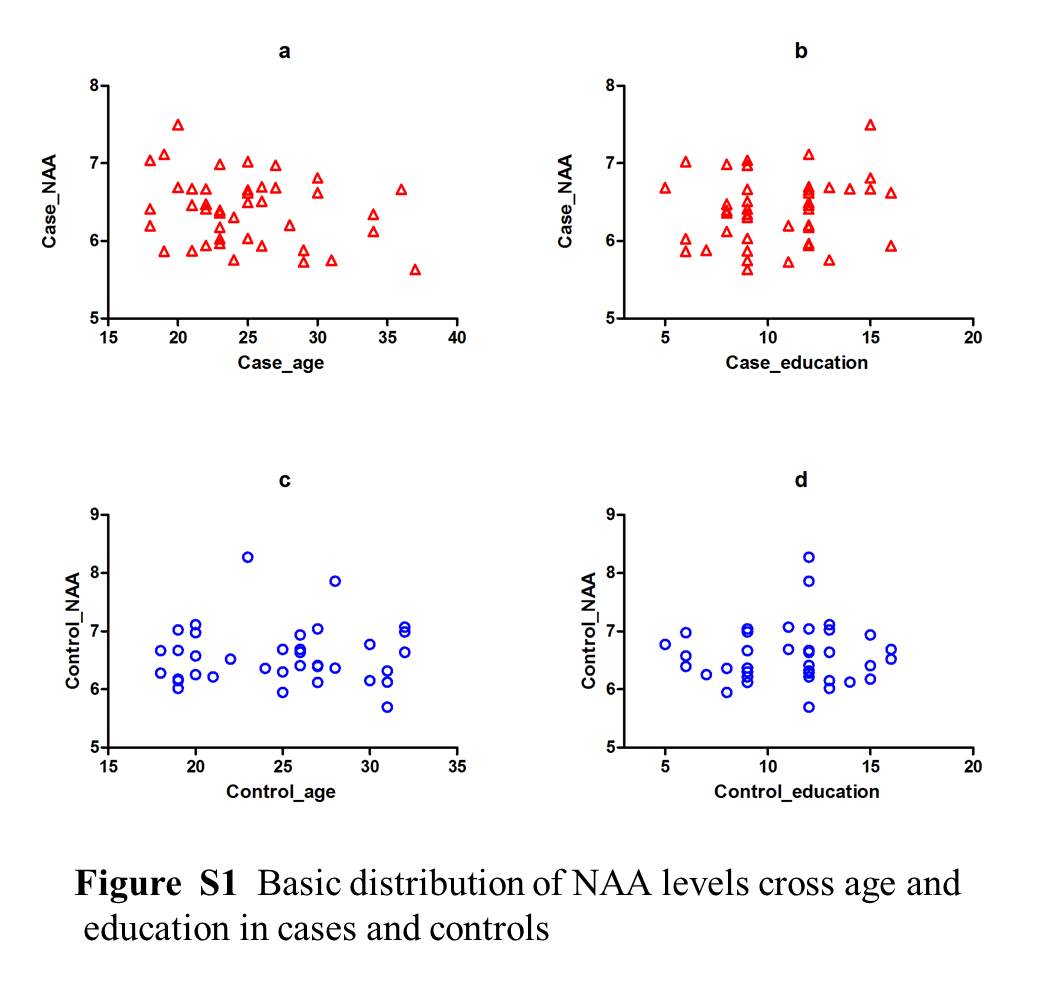
**

**Table S1. Comparison of baseline NAA, NAA/Cr+Pcr and Cr+Pcr among responders, non-responders and controls**

| **Metabolites** | **Control, n**=**38** | **Patient****s, n**=**42** | | **F** | **P** |
| --- | --- | --- | --- | --- | --- |
|  |  | Responders^a^  n=10 | Non-responders^b^  n=32 |  |  |
| NAA | 6.581±0.504 | 6.465±0.216 | 6.376±0.487 | 1.657 | 0.197 |
| NAA/Cr+Pcr | 1.432±0.098 | 1.428±0.197 | 1.409±0.125 | 0.314 | 0.732 |
| Cr+Pcr | 4.607±0.354 | 4.595±0.546 | 4.548±0.399 | 0.197 | 0.822 |
| Metabolites | Control, n=38 | Responders^c^  n=34 | Non-responders^d^  n=8 | F | P |
| NAA | 6.581±0.504 | 6.374±0.411 | 6.494±0.559 | 1.730 | 1.840 |
| NAA/Cr+Pcr | 1.432±0.098 | 1.421±0.143 | 1.380±0.148 | 0.586 | 0.559 |
| Cr+Pcr | 4.607±0.354 | 4.520±0.447 | 4.726±0.332 | 1.029 | 0.362 |

We divided the 42 patients into two kinds of groups based on their PANSS reductive ratio. ^a^PANSS reductive ratio≥0.5; ^b^PANSS reductive ratio<0.5; ^c^PANSS reductive≥0.25; ^d^PANSS reductive<0.25; PANSS reductive ratio=PANSS total scores (before treatment) – PANSS total scores (after treatment) / PANSS total scores (before treatment) – 30.

**Table S2. Longitudinal alterations of NAA and NAA/Cr+Pcr following 8-week treatment between responders and non-responders**

| **Metabolites** | **Patients, n**=**42** | | **t** | **P** |
| --- | --- | --- | --- | --- |
|  | Responders^a^  n=10 | Non-responders^b^  n=32 |  |  |
| NAA | 6.465±0.216 | 6.376±0.487 | 0.244 | 0.809 |
| NAA/Cr+Pcr | 1.428±0.197 | 1.409±0.125 | 0.363 | 0.719 |
| Metabolites | Responders^c^  n=34 | Non-responders^d^  n=8 | F | P |
| NAA | 6.374±0.411 | 6.494±0.559 | 1.392 | 0.172 |
| NAA/Cr+Pcr | 1.421±0.143 | 1.380±0.148 | 1.220 | 0.230 |

We divided the 42 patients into two kinds of groups based on their PANSS reductive ratio. ^a^PANSS reductive ratio≥0.5; ^b^PANSS reductive ratio<0.5; ^c^PANSS reductive≥0.25; ^d^PANSS reductive<0.25. PANSS reductive ratio=PANSS total scores (before treatment) – PANSS total scores (after treatment) / PANSS total scores (before treatment) – 30.
